# Supplementary material for: Quantitative and temporal analysis of autophagy: Differential Response to amino acid and glucose starvation
Source: PLoS One. 2026 Feb 4;21(2):e0340957. doi: 10.1371/journal.pone.0340957 (PMC12872001; doi:10.1371/journal.pone.0340957)
Supplement: S1 Fig — This file contains unmodified raw images of immunoblots included in this study. (PDF) [file pone.0340957.s001.pdf]

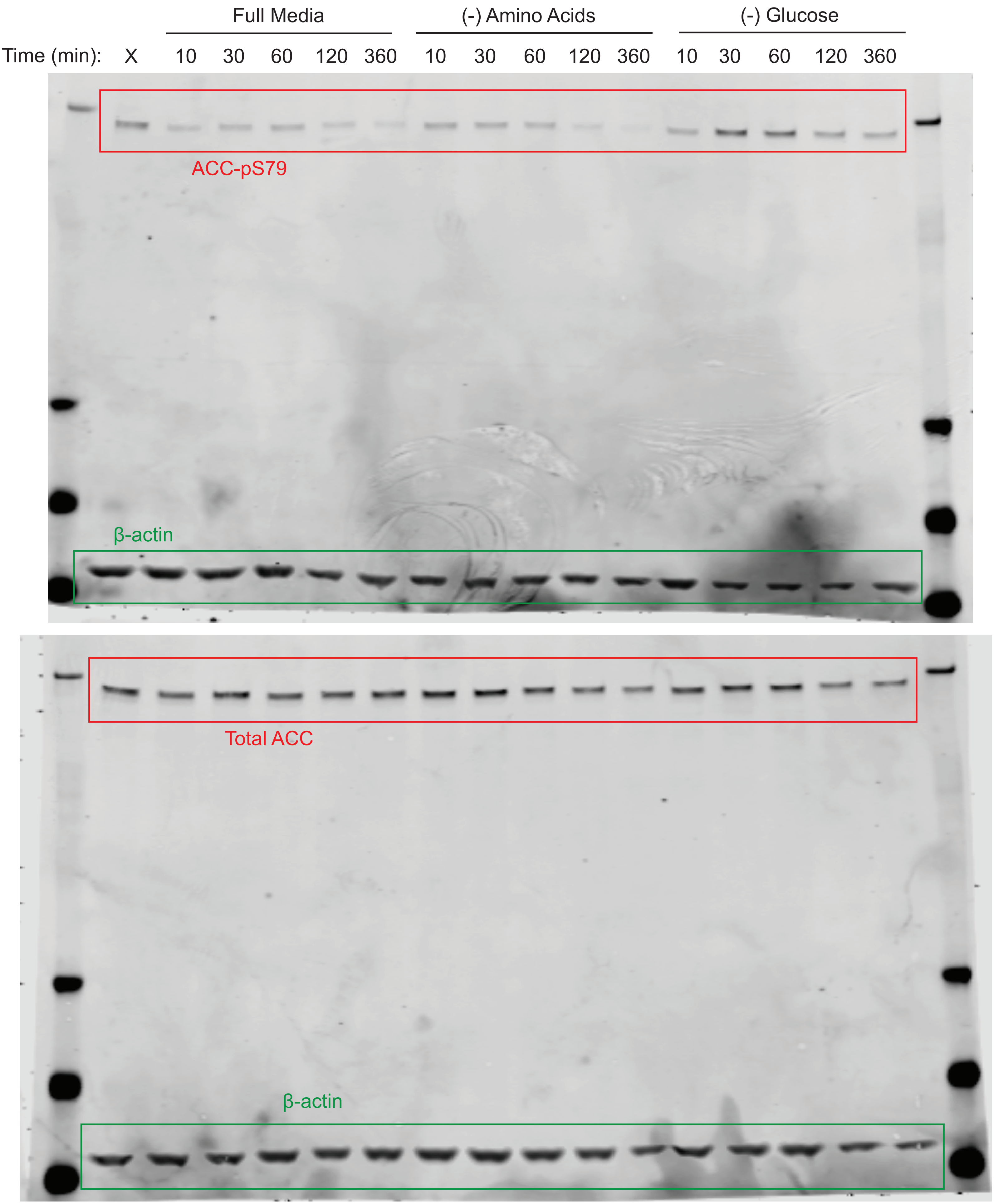

1st replicate

Related to Fig1

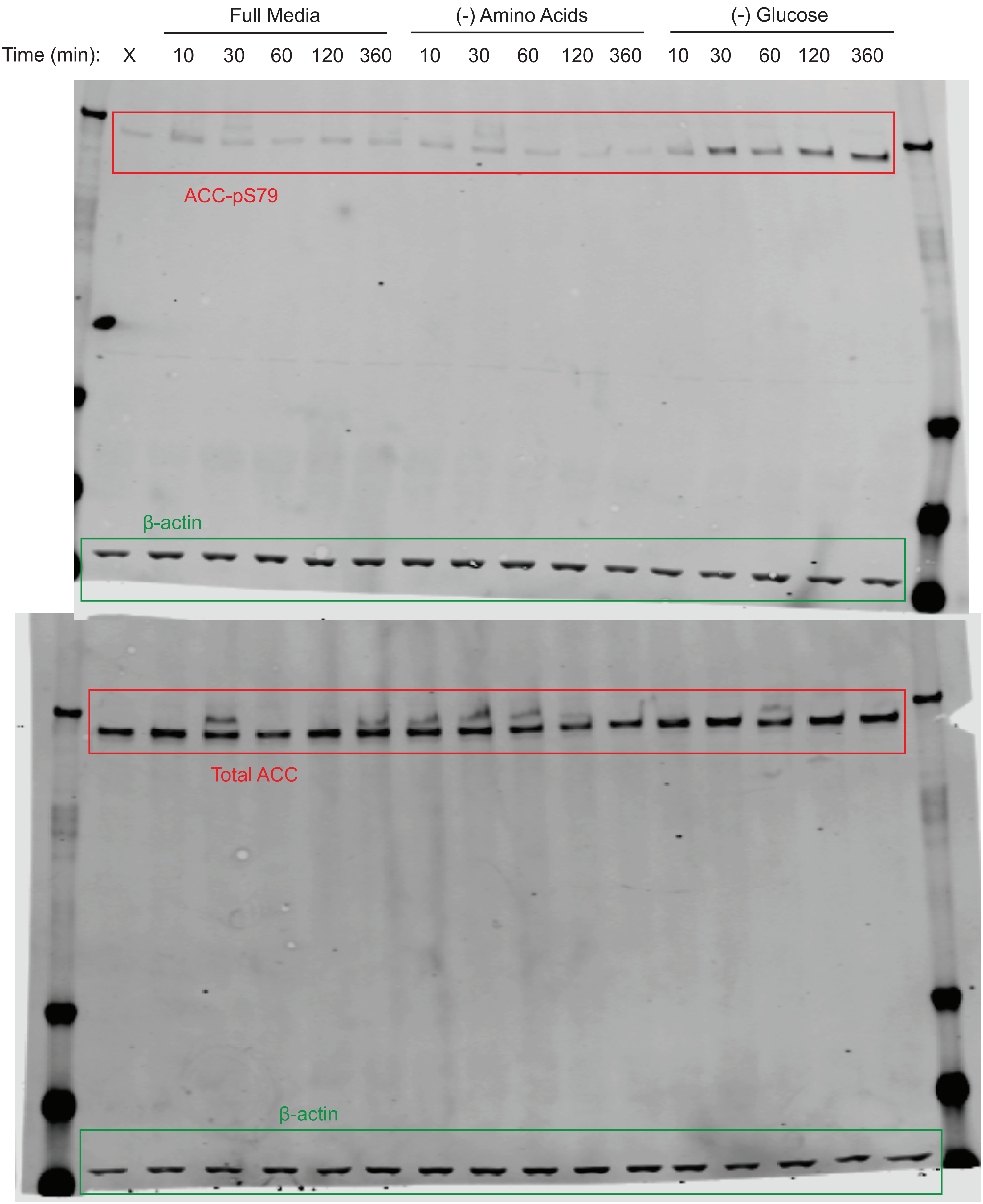

2nd replicate

Related to Fig1

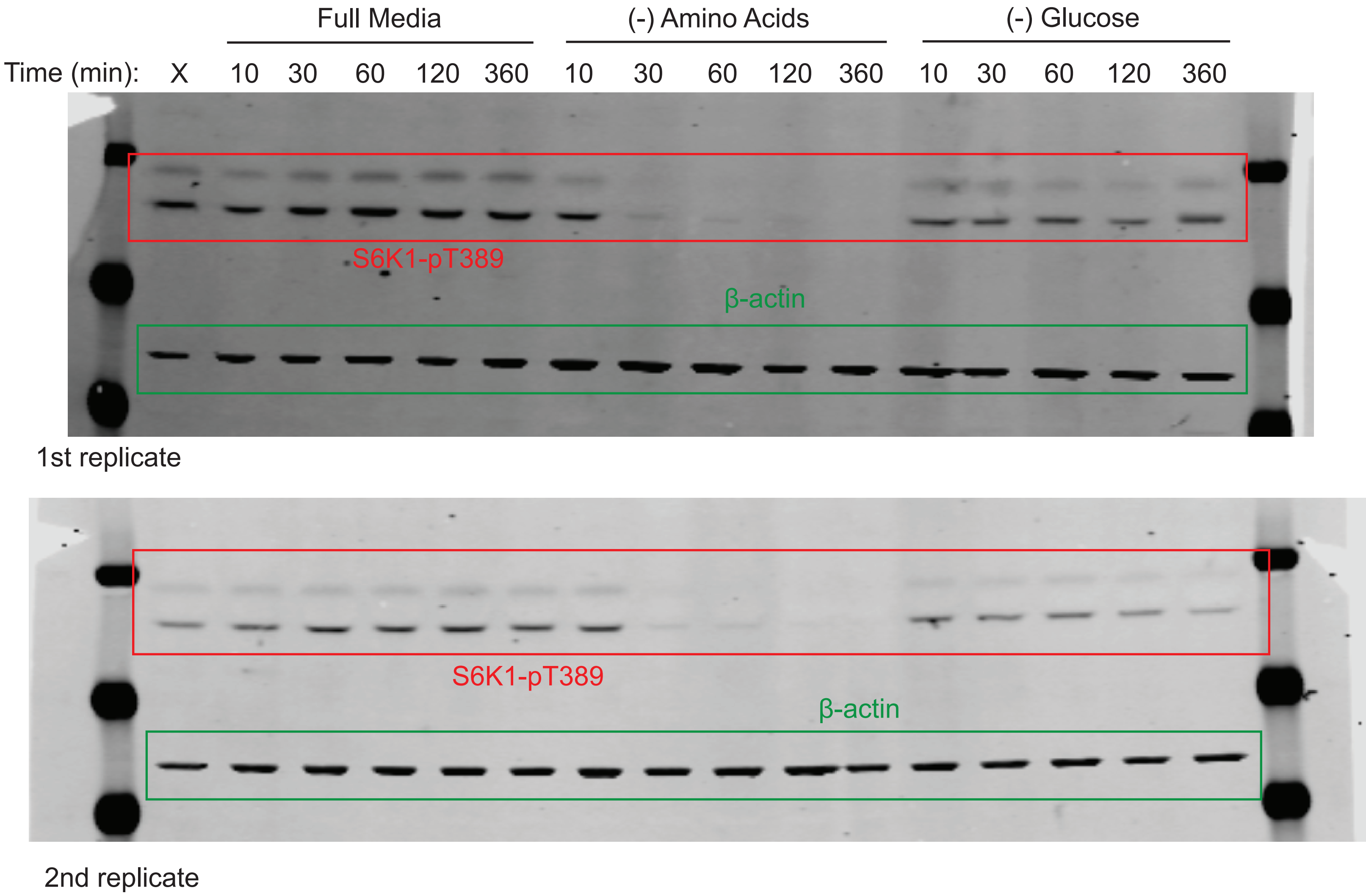

Related to Fig1

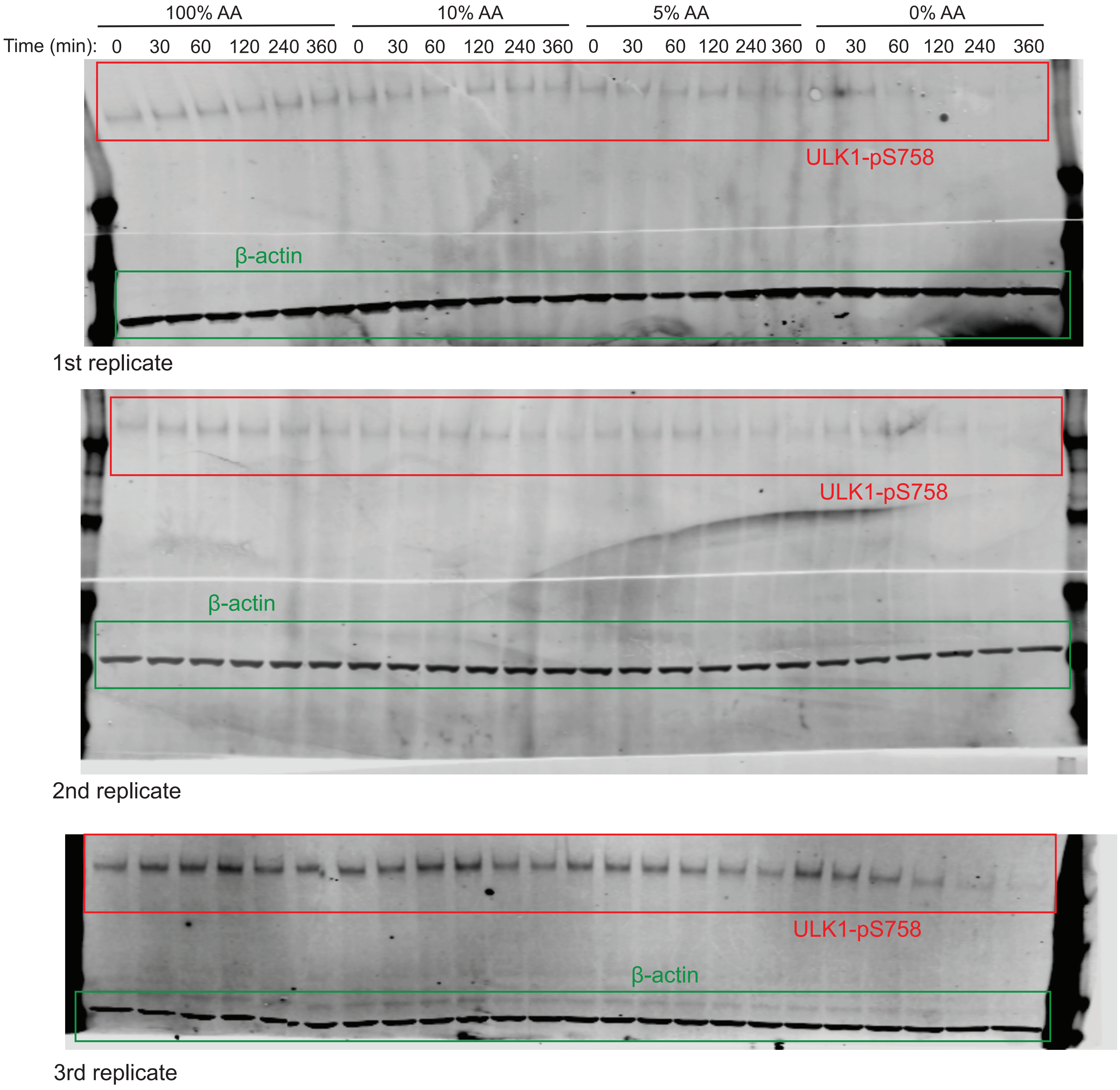

Related to Fig2

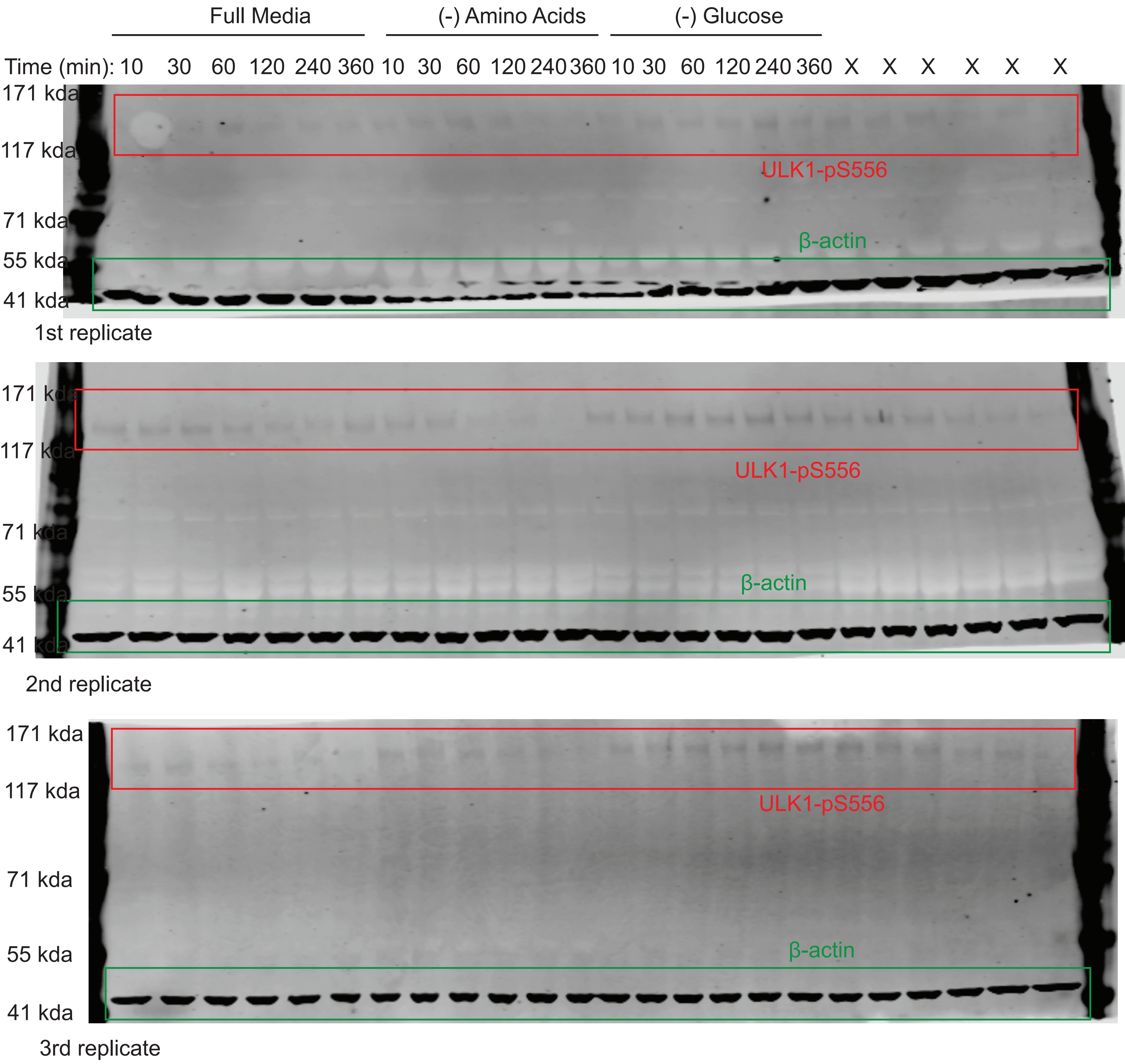

Related to FigS2

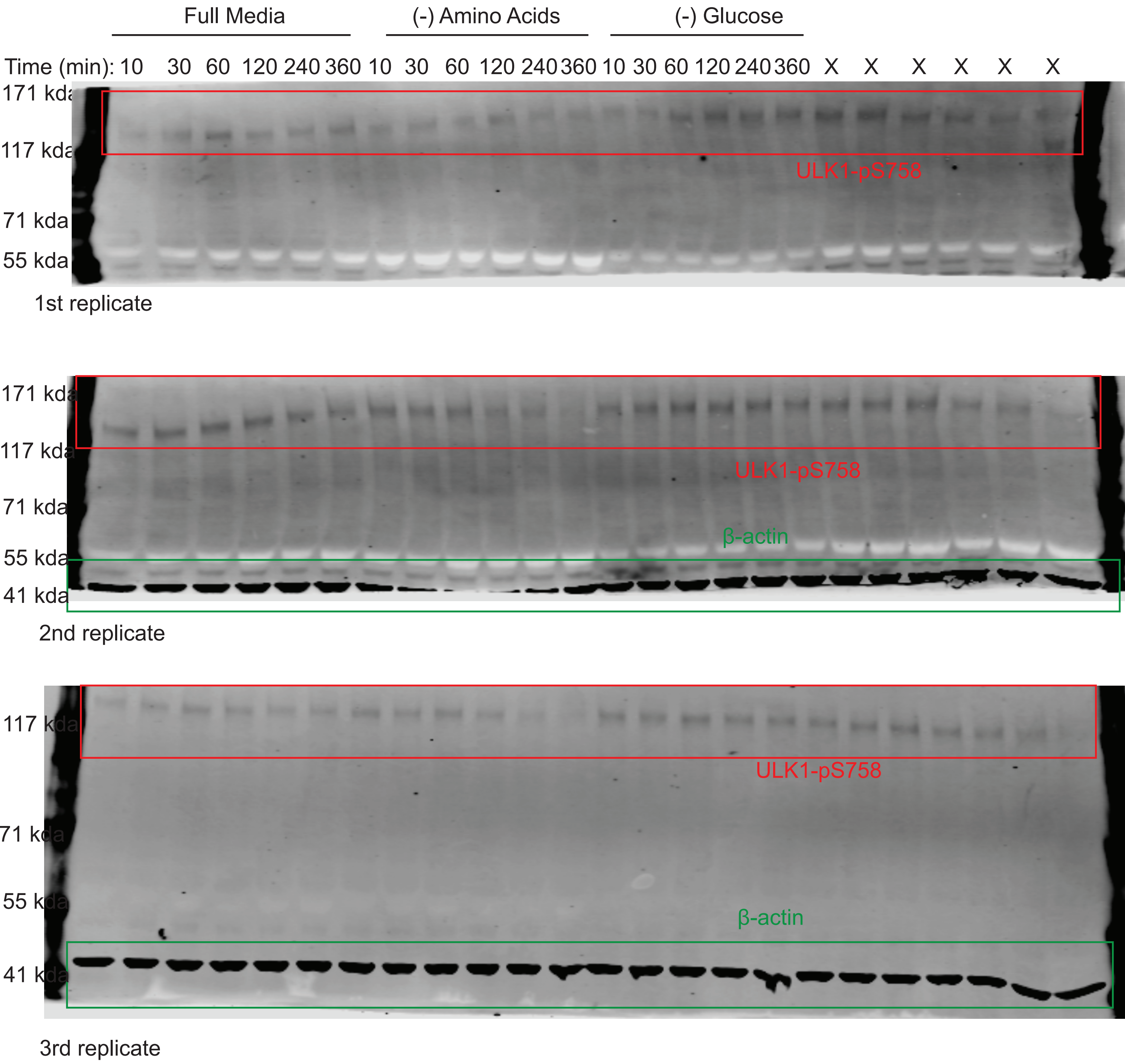

Related to FigS2
