# Supplementary figures and images for: Quantitative and temporal analysis of autophagy: Differential Response to amino acid and glucose starvation
Source: PLoS One. 2026 Feb 4;21(2):e0340957. doi: 10.1371/journal.pone.0340957 (PMC12872001; doi:10.1371/journal.pone.0340957)

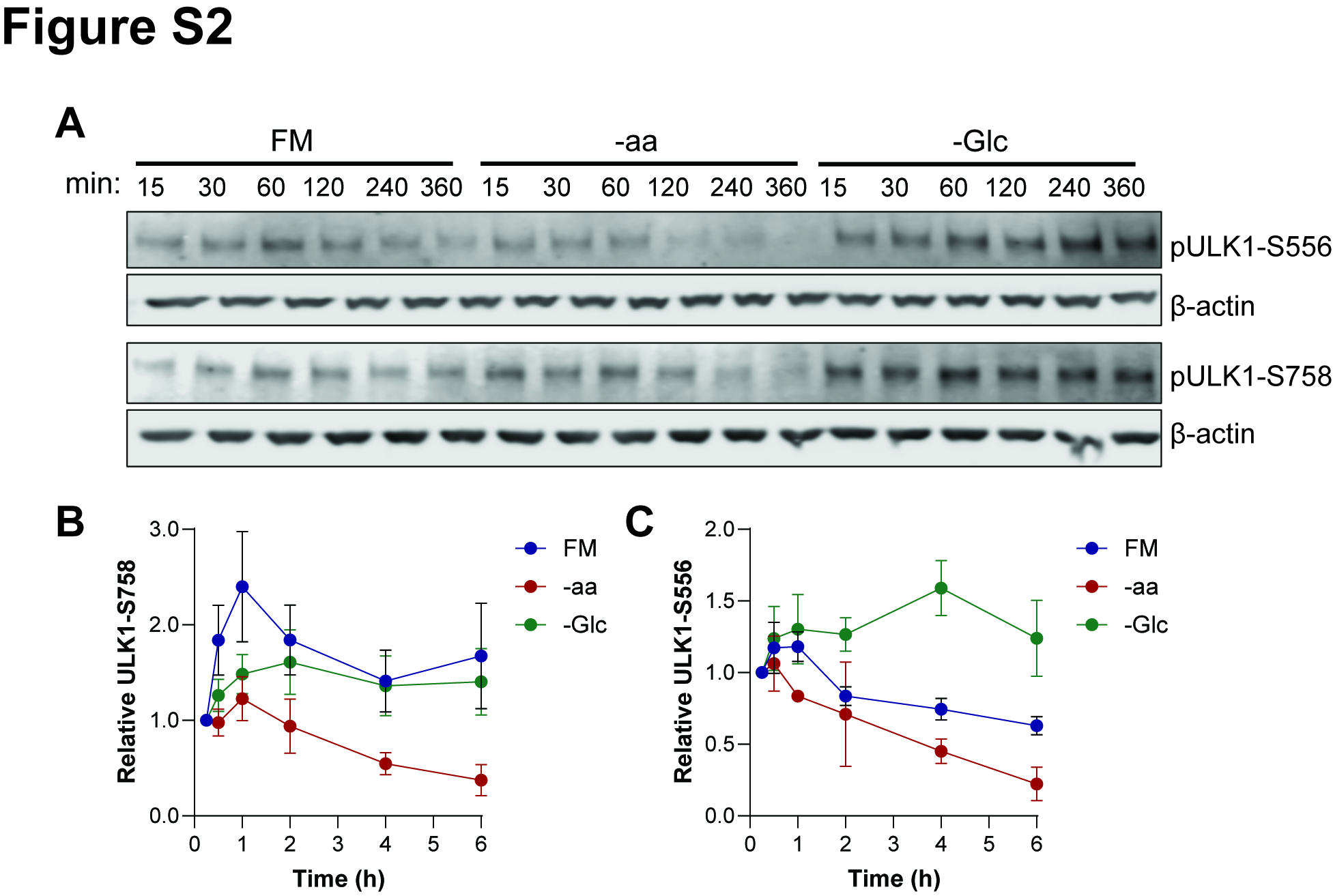

Supplement: S2 Fig — (A-C) U2OS cells were treated with full media (FM), aa-starvation media (-aa), or glucose-free media (-Glc) for the durations indicated before cells lysed and proteins analyzed by immunoblotting. A representative immunoblot is shown in (A). pULK1-S758 (relative to beta-actin) (B) or pULK1-S556 (relative to beta-actin) (C) were normalized to the first timepoint within each treatment and plotted with time (blue: FM; red: -aa; green: -Glc). Symbols are means of triplicate experiments and bars are s.e.m. (TIF) [file pone.0340957.s002.tif]
